# Phenotypes, Genotypes, Treatment, and Outcomes of 14 Children with Sitosterolemia at Vietnam National Children’s Hospital

**DOI:** 10.3390/jcm14020325

**Published:** 2025-01-07

**Authors:** Thi Thanh Mai Do, Chi Dung Vu, Tran Minh Dien, Thi Bich Ngoc Can, Thi Thanh Ngan Nguyen, Huy Hoang Nguyen, Van Khanh Tran, Ngoc Lan Nguyen, Huy Thinh Tran, Tran Thi Chi Mai, Khanh Ngoc Nguyen

**Affiliations:** 1 Hanoi Medical University, 1st Ton That Tung Street, Hanoi 11521, Vietnam; maidtt@nch.gov.vn (T.T.M.D.); dungvu@nch.gov.vn (C.D.V.); tranchimai@hmu.edu.vn (T.T.C.M.); 2 Center of Endocrinology, Metabolism, Genetic/Genomics and Molecular Therapy, Vietnam National Children’s Hospital, 18/879 La Thanh, Dong Da, Hanoi 11512, Vietnam; ngocctb@nch.gov.vn; 3 Vietnam National Children’s Hospital, 18/879 La Thanh, Dong Da, Hanoi 11512, Vietnam; dientm@nch.gov.vn; 4 Institute of Genome Research, Vietnam Academy of Science and Technology, 18 Hoang Quoc Viet Street, Cau Giay, Hanoi 100000, Vietnam; nganthanh27@yahoo.com (T.T.N.N.); nhhoang@igr.ac.vn (H.H.N.); 5 Center for Gene and Protein Research, Hanoi Medical University, 1st Ton That Tung Street, Hanoi 11521, Vietnam; tranvankhanh@hmu.edu.vn (V.K.T.); nguyenngoclan@hmu.edu.vn (N.L.N.); 6 Biochemistry Department, Hanoi Medical University, 1st Ton That Tung Street, Hanoi 11521, Vietnam; tranhuythinh@hmu.edu.vn

**Keywords:** sitosterolemia, hypercholesterolemia, *ABCG5*, *ABCG8*, xanthomas, Vietnamese patients

## Abstract

**Background**: Sitosterolemia is a rare autosomal recessive disorder characterized by diverse clinical manifestations ranging from asymptomatic cases to the development of xanthomas, hypercholesterolemia, premature atherosclerosis, or even sudden death during childhood. It results from homozygous or compound heterozygous pathogenic variants in the *ABCG5* or *ABCG8* genes. Prompt detection and intervention are essential to managing this condition and preventing severe outcomes. **Methods**: This study aims to retrospectively analyze the phenotype, genotype, treatment, and outcomes of 14 children—seven boys and seven girls—all of Vietnamese origin, diagnosed with sitosterolemia at the Vietnam National Children’s Hospital between March 2015 and July 2024. **Results**: The median ages at disease onset and diagnosis were 5.7 years (range: 1.5–17.9) and 7.2 years (range: 1.7–17.9), respectively. Xanthomas were observed in 85.7% of patients (12/14), arthralgia in 14.3% (2/14), and anemia in 7.1% (1/14), with no cases of thrombocytopenia. At diagnosis, all patients exhibited elevated total cholesterol (TC) and low-density lipoprotein cholesterol (LDL-C), with considerably higher levels in patients with xanthomas compared to those without. Mutations in the *ABCG5* gene were identified in 71.4% (10/14) of the patients, while 28.6% (4/14) had mutations in the *ABCG8* gene. Fourteen variants were detected, nine in *ABCG5* and five in *ABCG8*, with five variants reported for the first time in sitosterolemia patients. Initial management for all patients involved dietary modifications. After three months, 10 patients with persistently elevated TC and LDL-C received ezetimibe or cholestyramine treatment. Among the eight patients who continued treatment for over three months, the median TC and LDL-C concentrations decreased by 54.9% and 67.3%, respectively. **Conclusions**: Among Vietnamese patients with sitosterolemia, variants in the *ABCG5* gene were more prevalent than those in the *ABCG8* gene. Patients showed a positive response to ezetimibe or cholestyramine treatment. Genetic testing is essential for establishing a diagnosis of sitosterolemia and guiding accurate management strategies.

## 1. Introduction

Sitosterolemia (phytosterolemia) is a rare autosomal recessive disorder characterized by elevated plasma concentrations of plant sterols, xanthomas, and premature atherosclerosis [1]. Plant sterols and cholesterol are absorbed via Niemann-Pick C1 Like 1 (NPC1L1) and excreted through ABCG5 (sterolin 1) andABCG8 (sterolin 2) transporters [1]. Mutations in ABCG5 or ABCG8 lead to increased intestinal absorption and decreased biliary excretion of plant sterols and cholesterol, resulting in sitosterolemia [1]. Clinical manifestations vary widely, ranging from asymptomatic cases to significant phytosterol accumulation in plasma and organs, causing xanthomas, hypercholesterolemia, coronary atherosclerosis, and sudden death in childhood. Additional symptoms may include hemolytic anemia, thrombocytopenia, arthralgia, arthritis, splenomegaly, hepatomegaly, and liver failure [1,2,3,4,5,6,7].

The estimated frequency of homozygous or compound heterozygous pathogenic variants causing sitosterolemia is approximately 1 in 200,000 in the general population [8]. Sitosterolemia has been reported across various ethnic groups, including Spanish [4], Italian [5], Russian [9], Japanese [7,9], Korean [10], Brazilian [11], Colombian [12], and Saudi populations [13]. Pathogenic variants in the *ABCG5* or *ABCG8* genes, the transporter proteins ABCG5 and ABCG8, are responsible for the condition [14,15]. Case series have been documented in Chinese [3,16,17,18] and Japanese patients [15]. Previous studies have suggested that most Chinese probands with sitosterolemia have mutations in the *ABCG5* gene, whereas mutations in the *ABCG8* gene are more prevalent among probands of Caucasian origin [19,20].

Diagnosis of sitosterolemia, often based on the presence of xanthomas, premature coronary artery disease, and elevated phytosterol levels, can be challenging due to its clinical overlap with familial hypercholesterolemia [21]. In several cases, sitosterolemia is misdiagnosed as familial hypercholesterolemia [22,23,24]. Phytosterol testing is not widely available in clinical laboratories, and elevated phytosterol levels may also occur in patients with hypercholesterolemia [21] or in those carrying a single heterozygous *ABCG5/ABCG8* variant [15,25]. Molecular analysis has emerged as a definitive tool to confirm the diagnosis of sitosterolemia [11,26].

Treatment for sitosterolemia involves a low-sterol diet, intestinal cholesterol absorption inhibitors like ezetimibe, or bile acid sequestrant cholestyramine [8]. Unlike familial hypercholesterolemia, where statins are the first-line treatment [27], ezetimibe or cholestyramine is preferred for sitosterolemia. Early diagnosis and timely therapeutic interventions significantly improve prognosis and help prevent life-threatening cardiovascular events [1,3,28].

This study presents the clinical, biochemical, and molecular genetic characteristics and outcomes of 14 Vietnamese children with sitosterolemia from 12 unrelated families.

## 2. Materials and Methods

### 2.1. Subjects

We enrolled 14 patients diagnosed with sitosterolemia, who were followed at the Vietnam National Children’s Hospital from March 2015 to July 2024. Twelve probands younger than 16 years showed xanthomas, total cholesterol (TC) > 6.7 mmol/L, or low-density lipoprotein cholesterol (LDL-C) > 4 mmol/L. The probands were suspected of having recessive hypercholesterolemia. Molecular analysis identified that the probands had homozygous or compound heterozygous variants in the *ABCG5* or *ABCG8* genes. Two siblings without xanthomas were detected through family screening.

### 2.2. Clinical Analysis

Demographic data, family history, age at onset, age at diagnosis, clinical symptoms, and laboratory findings were collected for each patient. Routine blood lipid profiles included measurements of total cholesterol (TC), low-density lipoprotein cholesterol (LDL-C), high-density lipoprotein cholesterol (HDL-C), triglycerides, glutamic oxaloacetic transaminase (GOT), glutamic pyruvic transaminase (GPT), hemoglobin (Hb), and platelets.

### 2.3. Genetic Analysis

Genomic DNA was extracted from whole blood samples using the QIAamp DNA Blood Kit (Qiagen, Hilden, Germany). Genetic screening was conducted at Invitae (Invitae Corporation, San Francisco, CA, USA) using a comprehensive lipidemia panel that comprised the following 36 genes: *ABCA1* (NM_005502.3), *ABCG5* (NM_022436.2), *ABCG8* (NM_022437.2), *ANGPTL3* (NM_014495.3), *APOA1* (NM_000039.2), *APOA4* (NM_000482.3), *APOA5* (NM_052968.4), *APOB* (NM_000384.2), *APOC2* (NM_000483.4), *APOC3* (NM_000040.1), *CETP* (NM_000078.2), *CREB3L3* (NM_032607.2), *CYP7A1* (NM_000780.3), *CYP27A1* (NM_000784.3), *GALNT2* (NM_004481.4), *GCKR* (NM_001486.3), *GPD1* (NM_005276.3), *GPIHBP1* (NM_178172.5), *LCAT* (NM_000229.1), *LDLR* (NM_000527.4), *LDLRAP1* (NM_015627.2), *LIPA* (NM_000235.3), *LIPC* (NM_000236.2), *LIPG* (NM_006033.3), *LIPI* (NM_198996.3), *LMF1* (NM_022773.2), *LPL* (NM_000237.2), *LRP6* (NM_002336.2), *MTTP* (NM_000253.3), *MYLIP* (NM_013262.3), *PCSK9* (NM_174936.3), *PLTP* (NM_006227.3), *PNPLA2* (NM_020376.3), *SAR1B* (NM_001033503.2), *SCARB1* (NM_005505.4), and *ZHX3* (NM_015035.3). The genomic DNA underwent enrichment for targeted regions through a hybridization-based protocol and was sequenced using Illumina technology. The enrichment process focused on the coding sequences of the specified transcripts and included 20 base pairs of flanking intronic sequences. All targeted regions achieved sequencing coverage at a depth exceeding 50×. Reads were aligned to the hg19 reference sequence, and sequence changes were identified and interpreted in the context of a single clinically relevant transcript. Exonic deletions and duplications were detected using an in-house algorithm that calculated the copy number at each target by comparing the proband’s read depth with the mean read depth and distribution obtained from a clinical sample set.

Whole-exome sequencing (WES) was performed for patient S4 at the Institute of Genome Research. The WES and annotation processes followed previously established protocols [29]. Variant filtering was conducted across 308 genes (Supplementary Table S1) associated with abnormal circulating lipid concentrations (HPO_0003119, HPO annotations for rare diseases version 2024-01-11). Variants with a minor allele frequency > 0.01 were excluded, followed by the elimination of intronic variants. The zygosity of the remaining variants was assessed and compared with the inheritance pattern of the genes. Single heterozygous variants in recessive genes were excluded. Truncating mutations, frameshift insertions, and deletions were prioritized as high-impact variants. The pathogenicity of these variants was further evaluated using Combined Annotation Dependent Depletion (CADD) [30] and Mutation Taster [31] prediction tools.

### 2.4. Treatment, Follow-Up, and Outcomes

**Figure 1:**
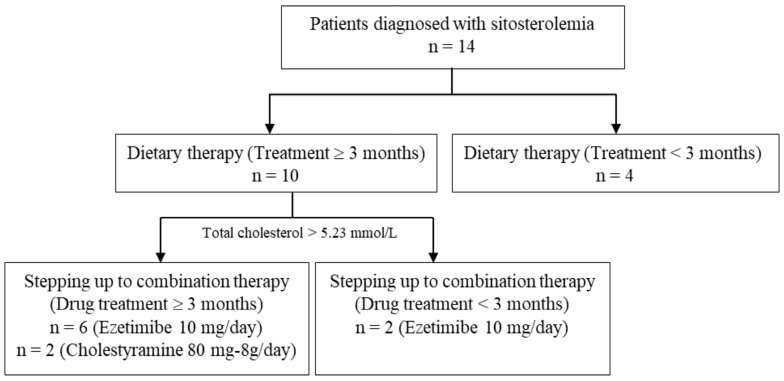
Flow diagram for treatment in our sitosterolemia cohort patients.

The treatment flow diagram is illustrated in Figure 1. Following diagnosis, all patients were placed on a specialized diet. The diets consisted of a maximum of 30% of total caloric intake from fat, with saturated fat limited to 7 calories and cholesterol restricted to less than 200 mg per day. Furthermore, patients had to refrain from consuming products rich in plant sterols, such as corn oil, sesame seeds, peanuts, soybeans, rapeseed oil, sesame oil, rice oil, margarine, avocado, chocolate, and shellfish. Alongside plant sterols, it was imperative to eschew foods high in cholesterol, such as animal liver and eggs. Patients consumed vegetables and fruits with lower levels of plant sterols, such as potatoes, carrots, and apples. After three months on the diet, 10 patients were evaluated for total cholesterol (TC) and low-density lipoprotein cholesterol (LDL-C) levels. Patients with TC > 5.23 mmol/L or LDL-C ≥ 3.3 mmol/L transitioned from dietary therapy to combination therapy with the intestinal cholesterol absorption inhibitor ezetimibe or cholestyramine. These ten patients were included in the treatment efficacy evaluation. Eight of the ten patients received combination therapy for a minimum of three months. Plasma TC and LDL-C concentrations were collected at three time points: the highest levels recorded before treatment, after three months of dietary therapy, and at the final visit prior to the treatment of sitosterolemia.

### 2.5. Statistical Analysis

Statistical analysis was conducted using SPSS version 26.0 software. Continuous variables were expressed as median (interquartile range), while categorical variables were presented as numbers (percentages). The Wilcoxon test was utilized for paired data, and the Mann–Whitney U test was applied for unpaired data to compare lipid levels at baseline with those following the intervention. A *p*-value of < 0.05 was considered statistically significant.

## 3. Results

### 3.1. Clinical Characteristics

Fourteen patients were diagnosed with sitosterolemia at the Centre for Endocrinology, Metabolism, Genetics/Genomics, and Molecular Therapy, Vietnam National Children’s Hospital, between March 2015 and July 2024. These patients originated from 12 unrelated families across eight provinces and comprised seven girls (50%) and seven boys (50%). The median age at onset was 5.7 years (range: 1.5–17.9 years) and the median age at diagnosis was 7.2 years (range: 1.7–17.9 years) (Table 1). Xanthomas were observed in 85.7% of patients (12/14), followed by arthralgia in 14.3% (2/14), and anemia in 7.1% (1/14). Xanthomas were found in the elbow joint, knee region, buttocks, ankle creases, wrists, and the extensor surface of the ankle joint. Two children (S2 and S14) were asymptomatic siblings identified through family screening.

All patients exhibited elevated levels of TC and LDL-C. Patients were categorized based on the presence of xanthomas and the type of variants in the *ABCG5* or *ABCG8* genes. The median TC and LDL-C levels in the 12 patients with xanthomas were 11.3 mmol/L (7.8–20.1 mmol/L) and 8.2 mmol/L (6.2–16.8 mmol/L), respectively, which were significantly higher than those in the two patients without xanthomas (*p* = 0.022) (Table 2). However, the median TC (11.8 mmol/L) and LDL-C (10.0 mmol/L) levels in the 10 patients with *ABCG5* variants were not significantly higher compared to the median TC (9.6 mmol/L) and LDL-C (7.3 mmol/L) levels in the four patients with *ABCG8* variants (*p* = 0.142 and *p* = 0.86, respectively).

### 3.2. Molecular Analyses

Ten patients (71.4%) harbored mutations in the *ABCG5* (NM_022436.2) gene, while four patients (28.6%) had mutations in the *ABCG8* (NM_022437.2) gene (Table 1). Among the 14 patients, four were homozygous for their respective variants (Table 1). A total of nine *ABCG5* variants and five *ABCG8* variants were identified (Table 1 and Table 3). These included five missense variants, four splicing variants, four nonsense variants, and one frameshift variant (Table 3), all predicted to be disease-causing by CADD and Mutation Taster tools. According to GnomAD, eight variants were reported in the heterozygous state in other ethnic groups, including *ABCG5* (c.331G>T, c.335dupA, c.751C>T, c.904+3_904+6del, c.1166G>A, and c.1711T>C) and *ABCG8* (c.694+5G>C and c.788G>A) (Table 1). One variant, *ABCG5*: c.1593C>A (p.Asn531Lys), was reported by Ambry Genetics in an individual with cardiovascular disease (Table 3). Three variants, *ABCG5*: c.1573C>T and *ABCG8*: c.687G>A and c.1757-2A>G, were previously identified by Invitae in sitosterolemia patients who were part of this study (Table 3). Two variants, *ABCG5*: c.403-2A>T and *ABCG8*: c.382A>T, have not been reported in the general population.

**Figure 2:**
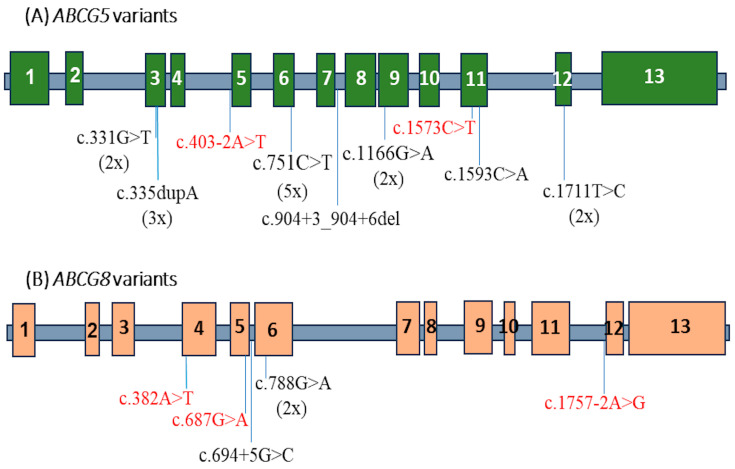
Schematic representation of identified variants in the *ABCG5* and *ABCG8* genes in 14 Vietnamese patients with sitosterolemia.

The nine *ABCG5* variants are distributed across exons 3, 6, 9, 11, and 12, as well as introns 4–5 and 7–8 (Figure 2A). The most frequent *ABCG5* variant, c.751C>T, was identified in five patients. The five *ABCG8* variants are located in exons 4, 5, and 6, and introns 5–6 and 11–12 (Figure 2B). The most common *ABCG8* variant, c.788G>A, was found in four patients.

### 3.3. Treatment and Outcomes

It is noted that before genetic analysis results were available, 13 patients in this cohort were initially misdiagnosed with heterozygous or homozygous familial hypercholesterolemia. Five patients—S4, S9, S10, S12, and S13—were treated with simvastatin but showed poor response. Additionally, patient S13 was treated with rosuvastatin (Crestor) at a dose of 20 mg/day, resulting in acute hepatitis symptoms, including elevated levels of liver enzymes (GOT (511.4 U/L), GPT (721.6 U/L), and GGT (339.3 U/L)) along with a decreased prothrombin ratio of 66%. These symptoms resolved one month after discontinuing Crestor treatment. 

Following genetic diagnosis, management for sitosterolemia patients was initiated. With dietary therapy alone for more than three months (n = 10), the median levels of TC and LDL-C were reduced compared to pre-treatment levels, but the changes were not statistically significant (p > 0.05) (Table 4). Some patients, including S2, S5, and S13, exhibited elevated TC and LDL-C levels during dietary therapy. All patients maintained higher than normal TC and LDL-C levels, necessitating escalation to combination therapy with ezetimibe or cholestyramine. Among these, eight patients who received drug treatment for over three months were selected for treatment efficacy evaluation. Results showed that these patients achieved normal TC levels (ranging from 2.96 to 5.02) and LDL-C levels (ranging from 1.92 to 3.36). The median TC and LDL-C levels significantly decreased by 54.9% and 67.3%, respectively (*p* = 0.012), compared to pre-treatment levels (Table 4).

## 4. Discussion

Twelve probands in our cohort with xanthomas and elevated TC and LDL-C levels were initially misdiagnosed with heterozygous or homozygous familial hypercholesterolemia. Notably, the LDL-C levels in some patients (S3, S4, S8, and S9) exceeded 13 mmol/L, the diagnostic threshold for homozygous familial hypercholesterolemia [14]. Although xanthomas were the most common symptom and initial presentation in hypercholesterolemia, their presence did not necessarily correlate with specific levels of hypercholesterolemia [37]. In this study, two patients, siblings of probands, did not present with xanthomas but were identified through biochemical testing, which confirmed hypercholesterolemia. Hematologic abnormalities, such as anemia and macrothrombocytopenia, are typical features that can differentiate sitosterolemia from familial hypercholesterolemia [8,18,38,39]. However, none of the probands in our study displayed hematologic abnormalities, except for patient S14, a sibling of proband S13, who had anemia (7.1%). This contrasts with reports from a small cohort of Chinese patients, where anemia accounted for up to 50% of cases [18]. Other features, such as splenomegaly and macrothrombocytopenia, commonly associated with sitosterolemia, were absent in our patients. Arthralgia was reported in two patients (14.3%), which is consistent with findings in Japanese cohorts [40].

Several studies have suggested that the diagnosis of sitosterolemia relies on clinical features. For instance, Zhang et al. proposed considering sitosterolemia in patients presenting with xanthomas, TC ≤ 15.41 mmol/L, LDL-C ≤ 13.22 mmol/L, mean platelet volume ≥ 9.05 fl, or Hb ≤ 120 g/L [18]. In our study, the diagnosis of sitosterolemia in 14 patients was confirmed based on the presence of disease-causing variants in the *ABCG5* or *ABCG8* genes, along with elevated levels of TC and LDL-C.

In our cohort, the proportions of patients with *ABCG5* and *ABCG8* variants were 71.4% and 28.6%, respectively. This finding aligns with several studies in the Chinese population [3,41], but differs from studies conducted in Iberoamerican countries [42]. The most common *ABCG5* variant identified in this study was c.751C>T (p.Gln251Ter), previously reported not only in Chinese patients [3,18], but also in an Italian patient [5]. Another common *ABCG5* variant, c.335dupA (p.Val113GlyfsTer85), was also observed in three Chinese patients [18]. Additionally, the c.1166G>A (p.Arg389His) variant in *ABCG5*, frequently found in Japanese patients [15] and Chinese patients [3,18,41], was noted. Among *ABCG8* variants, c.788G>A (p.Arg263Gln) was the most common in our study, consistent with findings in Chinese patients [41]. Notably, five variants, including *ABCG5*: c.403-2A>T and c.1573C>T, and *ABCG8*: c.382A>T, c.687G>A, and c.1757-2A>G, were documented for the first time in sitosterolemia patients. Of these, three nonsense variants—*ABCG5*: c.1573C>T (p.Gln525Ter), *ABCG8*: c.382A>T (p.Lys128Ter), and c.687G>A (p.Trp229Ter)—create premature stop codons, leading to absent or disrupted ABCG5/ABCG8 proteins. The two splicing variants, *ABCG5*: c.403-2A>T and *ABCG8*: c.1757-2A>G, likely affect pre-mRNA splicing by altering the acceptor splice site at the -2 position.

Correlation between genotype and phenotype has been reported in sitosterolemia. Kaya et al. observed significant differences in platelet levels, hemoglobin, and sitosterol concentrations between homozygous and heterozygous individuals [43]. However, in our study, no such correlation between genotype and phenotype was found. 

It is noteworthy that, prior to accurate diagnosis, our patients were initially misdiagnosed with familial hypercholesterolemia (FH). Five patients were treated with simvastatin but exhibited poor responses. Statins function by inhibiting the rate-limiting step of cholesterol synthesis in the liver through suppression of HMG-CoA reductase activity [44]. The poor response observed in our patients can be attributed to the fact that HMG-CoA reductase activity is already maximally inhibited in individuals with sitosterolemia. Additionally, patient S13, who received rosuvastatin (Crestor), had elevated liver enzyme levels. Previous studies have reported similar adverse effects of statins in sitosterolemia patients; for instance, increased liver enzyme levels were documented in French–Canadian patients [23]. Unfortunately, the misdiagnosis of FH resulted in delays in offering appropriate treatment to our patients. Initial treatments differ significantly between FH and sitosterolemia. In sitosterolemia, the first-line treatment involves intestinal cholesterol absorption inhibitors such as ezetimibe or bile acid sequestrants like cholestyramine [1,45]. In contrast, statins, as cholesterol synthesis inhibitors, are the first choice for treating FH [27].

After the definitive diagnosis of sitosterolemia, our patients were placed on a low-phytosterol diet for three months. However, most patients did not achieve agreeable responses to dietary therapy, and several patients showed elevated levels of TC and LDL-C. This outcome may be attributed to the difficulty children face in strictly adhering to their diet when consuming meals at kindergarten or school without parental supervision. Our findings align with those of previous studies, which demonstrated that a low-phytosterol diet reduced LDL plant sterols by 32% but had no significant effect on LDL cholesterol levels [46].

Subsequently, our patients were managed with ezetimibe or cholestyramine. After three months of combined dietary and ezetimibe/cholestyramine treatment, LDL-C levels normalized (<3.4 mmol/L) in all patients. Although ezetimibe is typically recommended for children aged 10 years and older, due to the risk of increased LDL-C and sterol levels associated with cardiovascular events and sudden death, four patients in our study under the age of 10 received ezetimibe treatment. These younger patients exhibited normal levels of GOT and GPT and no symptoms of arthralgia. In contrast to a prior study where two patients aged 10.6 and 58.9 years experienced arthralgia as a side effect of ezetimibe, our findings align with previous reports where younger patients under 10 years, including eight children aged three months to 7.8 years, were successfully treated with ezetimibe without adverse events [5,7,47,48,49,50]. The longest treatment duration reported was three years, except in one 11-month-old case who developed acute hepatitis when using a combination of ezetimibe (10 mg/day) and high-dose atorvastatin (40 mg/day). Following a medication pause, the child resumed ezetimibe at 10 mg/day, later combined with low-dose rosuvastatin (2.5 mg/day), without recurrence of hepatitis [49]. These findings highlight the need for long-term studies to evaluate the efficacy and safety of ezetimibe in children under 10 years of age.

Previous studies have suggested that heterozygous carriers of loss-of-function *ABCG5* variants exhibit elevated levels of sitosterol and LDL-C, which may increase their risk of coronary artery disease [11,33]. Therefore, we consulted other members of the patients’ families regarding these potential risks and recommended appropriate monitoring and preventive measures.

Plant sterol concentration is an important index for evaluating treatment effectiveness, prognosis, and disease screening. It is necessary to measure plant sterol levels in plasma using mass spectrometry. Sitosterolemia is diagnosed at serum sitosterol levels equal to or greater than 10.0 µg/mL [8]. Additionally, an association between sitosterolemia and hypercholesterolemia was reported [51]. About 27 of 100 patients with β-sitosterol concentrations ≥ 15.0 µg/mL presented with LDL-C values ≥ 4.91 mmol/L (190 mg/dL), whereas four of 100 patients with β-sitosterol concentrations < 8.0 µg/mL had LDL-C values ≥ 4.91 mmol/L (190 mg/dL) [51]. Unfortunately, we were unable to assess the patients’ sterol levels due to the unavailability of this testing in our hospital, which represents a limitation of our study. Consequently, medication treatment was guided solely by LDL-C levels, aiming to achieve LDL-C < 3.3 mmol/L or a 50% reduction from baseline.

## 5. Conclusions

Our study described the clinical characteristics, biochemical features, molecular analysis, treatment, and outcomes of 14 Vietnamese patients with sitosterolemia. Among Vietnamese patients with sitosterolemia, *ABCG5* gene variants were more common than *ABCG8* gene variants. The patients responded well to treatment with ezetimibe or cholestyramine and dietary therapy. Genetic testing facilitates the accurate diagnosis of sitosterolemia and aids in selecting appropriate management strategies for patients.

## Figures and Tables

**Table 1:** Clinical characteristics of sitosterolemia in 14 patients.

No	Sex	Age of Onset (Year)	Age of Diagnosis (Year)	Family History	Xanthomas	TC (mmol/L)	LDL-C (mmol/L)	Anemia	Thrombocytopenia	Arthralgia	Variant
S1	F	1.56	1.68	Sibling of S2	+	+11.77	+8.35	−	−	−	*ABCG5*: c.331G>T/c.751C>T
S2	M	2.68	2.72	Sibling of S1	−	+6.34	+4.39	−	−	−	*ABCG5*: c.331G>T/c.751C>T
S3	M	1.51	8.31	−	+	+17.21	+13.51	−	−	−	*ABCG5*: c.335dupA/c.335dupA
S4	M	4.52	4.56	−	+	+20.07	+14.68	−	−	−	*ABCG5*: c.335dupA/c.751C>T
S5	M	1.77	1.84	−	+	+6.09	+4.71	−	−	−	*ABCG5*: c.335dupA/c.1166G>A
S6	M	6.18	6.44	−	+	+10.89	+7.79	−	−	+	*ABCG5*: c.403-2A>T/c.751C>T
S7	F	7.50	7.86	−	+	+8.79	+6.15	−	−	−	*ABCG5*: c.751C>T/c.1711T>C
S8	M	1.49	1.78	−	+	+18.98	+16.79	−	−	−	*ABCG5*: c.904+3_904+6del/c.1593C>A
S9	F	5.30	5.35	−	+	+17.91	+13.02	−	−	−	*ABCG5*: c.1166G>A/c.1166G>A
S10	M	7.78	11.14	−	+	+17.27	+11.66	−	−	−	*ABCG5*: c.1573C>T/c.1711T>C
S11	F	10.94	11.08	−	+	+10.83	+6.97	−	−	−	*ABCG8*: c.382A>T/c.694+5G>C
S12	F	8.11	15.02	−	+	+9.67	+7.53	−	−	−	*ABCG8*: c.687G>A/c.1757-2A>G
S13	F	9.46	9.51	Sibling of S14	+	+9.50	+8.05	−	−	+	*ABCG8*: c.788G>A/c.788G>A
S14	F	17.89	17.93	Sibling of S13	−	+7.78	+6.29	+	−	−	*ABCG8*: c.788G>A/c.788G>A

F: Female; M: Male; LDL-C: Low-density lipoprotein cholesterol.

**Table 2:** Total cholesterol and low-density lipoprotein cholesterol in the xanthomas group compared with non-xanthomas and in the *ABCG5* group compared to the *ABCG8* group.

Parameter	Xanthomas			Variant		
	Present n = 12	None n = 2	*p*	*ABCG5* n = 10	*ABCG8* n = 4	*p*
TC (median) (mmol/L)	11.3 (7.8–20.1)	6.2 (6.1–6.3)	0.022	11.8 (6.3–20.1)	9.6 (6.1–10.8)	0.142
LDL-C (median) (mmol/L)	8.2 (6.2–16.8)	4.6 (4.4–4.7)	0.022	10.0 (4.4–16.8)	7.3 (4.7–8.1)	0.240

TC: Total cholesterol; LDL-C: Low-density lipoprotein cholesterol.

**Table 3:** *ABCG5* and *ABCG8* variants identified in 14 Vietnamese sitosterolemia patients.

Gene	c.DNA change	Amino Acid Change	CADD (Score)	Mutation Taster	dbSNP	ClinVar	GnomAD	Reported Patients
*ABCG5*	c.331G>T	p.Gly111Trp	Del	Disease	0	VUS 1981625 (Invitae)	1/1157852 (European)	This study
*ABCG5*	c.335dupA	p.Val113GlyfsTer85	Del	Disease	rs1470569820	Pathogenic 2616584	2/42584 (East Asian)	[18]
*ABCG5*	c.403-2A>T	Splicing	Del	Disease	0	0	0	This study
*ABCG5*	c.751C>T	p.Gln251Ter	Del	Disease	rs140111105	Pathogenic 1120119	12/44888 (East Asian)	[3,5,18]
*ABCG5*	c.904+3_904+6del	Splicing	Del	Disease	rs1167123880	VUS 1980073 (Invitae)	1/44860 (East Asian)	This study
*ABCG5*	c.1166G>A	p.Arg389His	Del	Disease	rs119480069	Pathogenic 4980	72/44882 (East Asian)	[3,15,18,32,33,34]
*ABCG5*	c.1573C>T	p.Gln525Ter	Del	Disease	0	Pathogenic 2023964 (Invitae)	0	This study
*ABCG5*	c.1593C>A	p.Asn531Lys	Del	Disease	0	VUS 1939772 (Invitae+Ambry Genetics)	0	This study
*ABCG5*	c.1711T>C	p.Cys571Arg	Del	Disease	rs370371131	VUS 595767	2/74808 (African)	This study
*ABCG8*	c.382A>T	p.Lys128Ter	Del	Disease	0	0	0	This study
*ABCG8*	c.687G>A	p.Trp229Ter	Del	Disease	rs141909291	Pathogenic 2117818 (Invitae)	0	This study
*ABCG8*	c.694+5G>C	Splicing	Del	Disease	rs557890655	VUS 593563	96/44870 (East Asian)	[3,15,32]
*ABCG8*	c.788G>A	p.Arg263Gln	Del	Disease	rs137852990	VUS 4970	34/44886 (East Asian)	[19,35,36]
*ABCG8*	c.1757-2A>G	Splicing	Del	Disease	0	Likely pathogenic 2117820 (Invitae)	0	This study

CADD, combined annotation-dependent depletion; Del, deleterious; VUS, variant of uncertain significance.

**Table 4:** Treatment and outcomes of 10 sitosterolemia patients.

Treatment	Variables	Pre-Treatment	Post-Treatment	*p*
Phase I: Dietary treatment > 3 months (n = 10)	TC (mmol/L)	10.7 (6.1−18.9)	8.4 (6.5−12.6)	0.093
	LDL-C (mmol/L)	8.2 (4.4−16.8)	6.1 (4.5−9.2)	0.059
Phase II: Drug treatment > 3 months Ezetimible/Cholestyramin + dietary (n = 8)	TC (mmol/L)	8.4 (6.5−12.6)	4.3 (2.7−5.0)	0.012
	LDL-C (mmol/L)	6.1 (4.5−9.2)	2.6 (1.9−3.4)	0.012

TC, total cholesterol; LDL-C, low-density lipoprotein cholesterol.

## Data Availability

The original contributions presented in this study are included in the article/Supplementary Material. Further inquiries can be directed to the corresponding author(s).

## Supplementary Materials

The following supporting information can be downloaded at: https://www.mdpi.com/article/10.3390/jcm14020325/s1, Table S1: List of genes associated with abnormal circulating lipid concentrations.
